# Association between IQ and *FMR1* protein (FMRP) across the spectrum of CGG repeat expansions

**DOI:** 10.1371/journal.pone.0226811

**Published:** 2019-12-31

**Authors:** Kyoungmi Kim, David Hessl, Jamie L. Randol, Glenda M. Espinal, Andrea Schneider, Dragana Protic, Elber Yuksel Aydin, Randi J. Hagerman, Paul J. Hagerman

**Affiliations:** 1 UC Davis MIND Institute, UC Davis Health, Sacramento, California, United States of America; 2 Department of Public Health Sciences, University of California, Davis, School of Medicine, Davis, California, United States of America; 3 Department of Psychiatry and Behavioral Sciences, University of California, Davis, School of Medicine, Sacramento, California, United States of America; 4 Department of Biochemistry and Molecular Medicine, University of California, Davis, School of Medicine, Davis, California, United States of America; 5 Department of Pediatrics, University of California, Davis, School of Medicine, Sacramento, California, United States of America; Centre National de la Recherche Scientifique, FRANCE

## Abstract

Fragile X syndrome, the leading heritable form of intellectual disability, is caused by hypermethylation and transcriptional silencing of large (CGG) repeat expansions (> 200 repeats) in the 5′ untranslated region of the fragile X mental retardation 1 (*FMR1*) gene. As a consequence of *FMR1* gene silencing, there is little or no production of *FMR1* protein (FMRP), an important element in normal synaptic function. Although the absence of FMRP has long been known to be responsible for the cognitive impairment in fragile X syndrome, the relationship between FMRP level and cognitive ability (IQ) is only imprecisely understood. To address this issue, a high-throughput, fluorescence resonance energy transfer (FRET) assay has been used to quantify FMRP levels in dermal fibroblasts, and the relationship between FMRP and IQ measures was assessed by statistical analysis in a cohort of 184 individuals with CGG-repeat lengths spanning normal (< 45 CGGs) to full mutation (> 200 CGGs) repeat ranges in fibroblasts. The principal findings of the current study are twofold: i) For those with normal CGG repeats, IQ is no longer sensitive to further increases in FMRP above an FMRP threshold of ~70% of the mean FMRP level; below this threshold, IQ decreases steeply with further decreases in FMRP; and ii) For the current cohort, a mean IQ of 85 (lower bound for the normal IQ range) is attained for FMRP levels that are only ~35% of the mean FMRP level among normal CGG-repeat controls. The current results should help guide expectations for efforts to induce *FMR1* gene activity and for the levels of cognitive function expected for a given range of FMRP levels.

## Introduction

Fragile X syndrome (FXS) is the leading heritable form of intellectual disability and the leading monogenic form of autism spectrum disorder (ASD). In addition to cognitive impairment, individuals with FXS can manifest a broad range of behavioral and psychiatric symptoms (e.g., hyperactivity, impulsivity, aggression, and anxiety), poor language development, seizures, and characteristic physical features (see Reviews [1, 2–4]). In nearly every instance, FXS is caused by expansion of a 5′ noncoding trinucleotide (CGG) beyond ~200 repeats (full mutation; FM) in the fragile X mental retardation 1 (*FMR1*) gene. These large CGG-repeat expansions generally lead to hypermethylation of the promoter and CGG-repeat regions, with attendant transcriptional silencing and little or no expression of the *FMR1* protein (FMRP) [5–8]. FMRP, through its capacity as an RNA binding protein [9–13], plays a critical role in neuronal function by regulating the translation, transport, and stabilization of a substantial number of mRNAs involved in the development and maintenance of synaptic connections [11, 14–22], and through its role in the homeostatic control of systemic metabolism [1, 23]. FMRP has also been shown to play a role in both the DNA damage response and the maintenance of genome stability [24, 25], and in the regulation of ion channel activity through its direct interactions with ion channel proteins [26–28]. Reduction or absence of FMRP, with resultant effects on brain morphology and function [29–31], is thought to be directly responsible for the cognitive and behavioral problems that are core features of the FXS phenotype [1].

For those whose *FMR1* gene retains at least partial activity, the association between the severity of intellectual disability and the degree of FMRP deficit remains ill-defined. For example, ample evidence shows that FMRP levels are moderately diminished in both peripheral and CNS cells that harbor *FMR1* alleles in the premutation range (55–200 CGG repeats), in part due to reduced efficiency of translation of the expanded-repeat *FMR1* mRNA [32–34] and due to the increased likelihood that the larger premutation alleles may be at least partially methylated [35]. Thus, reductions in FMRP, if of sufficient magnitude, could result in some degree of intellectual deficit even within the premutation range [32, 36, 37]. It is also unclear to what extent unmethylated FM alleles, which are capable of producing substantial quantities of *FMR1* mRNA [38, 39], have the capacity to produce FMRP from the expanded-repeat mRNA due to the inefficiency of translation from RNAs containing FM CGG repeats [8]. Early studies of individual fibroblast clones or lymphoblastoid lines suggested that FM alleles above ~300 CGG (either methylated or unmethylated) do not express FMRP [8, 14, 40, 41]. Kaufmann et al. [42] also reported low FMRP levels (10.3 ± 1.7% of normal levels) for subjects with FM alleles, although the FM CGG-repeat sizes were not reported. More recently, Pretto et al. [43] observed that males with a fully methylated FM allele had essentially no FMRP expression (by Western blot analysis). In one of their cases, a size- and methylation-mosaic male with a methylated 180 CGG-repeat allele and unmethylated ~200–270 CGG-repeat alleles had substantial FMRP, reinforcing the idea that FM alleles under ~300 CGG repeats, if unmethylated, are still capable of producing some FMRP.

Confounding the association between FMRP levels and intellectual deficit are numerous reports of males described as being of average to above average intellectual level, or mildly affected, with partially methylated alleles in the FM range [44–54]. However, part of the difficulty in assessing the relationship between IQ and genotype/FMRP expression has been distinguishing individuals who harbor exclusively FM alleles from those who possess both FM and premutation (PM; 55–200 CGG repeats) alleles. Indeed, newer studies have revealed that the majority of individuals with FM alleles are mosaics for allele size, harboring both unmethylated PM alleles and FM alleles [55–58], with such individuals retaining the capability of producing at least some FMRP. The other confound associated with studies of FMRP after 1995 has been the use of the immunocytochemical method [59] to assess the presence/absence of FMRP. Although the immunocytochemical method remains perfectly valid as a measure of fractional inactivation (e.g., gene deletion; activation ratio in females), its use as a surrogate measure for relative FMRP level (i.e., % FMRP(+) cells ≡ FMRP relative level) can lead to large uncertainties in FMRP levels because FMRP levels within each cell are not quantitated.

The question of how much *FMR1* gene activity–and FMRP production–is possible in the FM range is of critical importance in determining whether efforts to reactivate FM alleles [60–69] will ultimately succeed in producing enough FMRP to have a positive effect on brain and cognitive function. However, the more immediate question, and the focus of the current study, concerns the relationship between cognitive ability and FMRP levels, irrespective of CGG-repeat size: how much FMRP is necessary for borderline to average intellectual functioning? The relevance of this question extends beyond the fragile X-associated disorders to other forms of neurodevelopmental and neuropsychiatric dysfunction, as exemplified by lowered FMRP levels in the brains of those with autism/ASD [70–72] and major psychiatric disorders, including depression and schizophrenia [73, 74].

More recently, several approaches have been developed to provide more quantitative estimates of FMRP levels from human cells/tissues. Iwahashi et al. [75] utilized an enzyme-linked immunosorbent assay (ELISA) for FMRP. Using this assay, the range of FMRP detected in the subjects with hypermethylated full-mutation alleles was 0.48% to 4.45% (*n* = 6) of levels observed in control males. The highest FMRP value (4.45%) corresponded to a subject with one of the smallest FM alleles (285 CGG repeats), adding support to the notion that only alleles under ~300 CGG repeats are capable of producing significant amounts of FMRP. Iwahashi et al. [76] also observed slightly higher FMRP levels (1.07% to 13.02% of control mean levels), where the higher FMRP levels could be based on smaller unmethylated FM alleles (<300 CGG repeats), as well as contributions from PM alleles. In aggregate, previous studies [58, 77, 78] indicate that significant FMRP expression arises primarily from PM alleles and not from FM alleles of greater than ~300 CGG repeats, regardless of methylation. Therefore, in discussing cases of high IQ with moderate FMRP levels, it is highly likely that individuals may be harboring size-mosaic *FMR1* alleles.

In the current work, we have applied the method of fluorescence resonance energy transfer (FRET) [69, 79] to the study of the relationship between FMRP level and IQ in 184 cases with CGG lengths spanning from normal to the FM range. We observed an FMRP-level threshold, at ~70% of the mean value for those with normal CGG repeats, above which IQ no longer depends on FMRP level. For FMRP levels that exceed ~35% of the mean FMRP level among those in the normal CGG-repeat range, the upper 95%-ile for IQ values for the current cohort approaches 85 (lower bound for normal IQ range), suggesting that this level of FMRP may be sufficient for supporting its role in attaining normal intellectual functioning.

## Materials and methods

### Subjects

The Institutional Review Board of the University of California, Davis, approved all study protocols and written informed consent forms associated with this research. Written consent was obtained from study subjects. The subjects of this study initially included 195 individuals (121 males and 74 females; 161 white, 1 black, 5 Hispanic/Latino, 4 multi-ethnic, 2 Asian, 2 others, and 20 unknown) recruited to the University of California Davis Health MIND Institute’s Fragile X Clinical and Research Center between 2007 and 2018 as participants in studies of fragile X-associated disorders. Eleven individuals (9 males and 2 females) were subsequently excluded based on secondary, known genetic neurodevelopmental disorders, a point mutation [80], and/or dysmorphic features atypical for FXS; all of those individuals had FMRP levels within the normal range (the point mutation was detected by Western blot but not by FRET assay) but were not used for further analyses.

Full thickness skin biopsies from the left posterior shoulder area were obtained with a 3 mm punch under local anesthesia with lidocaine. The biopsies were placed in RPMI 1640 (Gibco) culture media, stored at 4°C, and processed within 24 hours. The samples were diced into approximately 15 pieces under sterile conditions on a culture plate. The pieces were distributed among three culture dishes containing 60:40 RPMI 1640:Amnio MAXTM-C100 Basal Medium (Gibco, Grand Island, NY, USA) and 15% AmnioMAXTM-C100 Supplement (Gibco). Fibroblasts were allowed to grow out from the tissue pieces for 2–3 weeks, followed by growth for no more than three passages. Cells were frozen down using 10% dimethyl sulfoxide (DMSO) in 60:40 media until needed.

### IQ measures

Intellectual functioning was measured using the Wechsler Intelligence Scale for Children, Third or Fourth Edition (WISC-III or WISC-IV; Pearson, London), or the Stanford Binet Intelligence Scales, Fifth Edition (SB-5; Harcourt, San Antonio), for children ages 8–17 years, and using the Wechsler Adult Intelligence Scale, Third or Fourth Edition (WAIS-III or WAIS-IV), or the SB-5 for adults ages 18 years and up. As the assessments spanned many protocols and years of data collection, multiple test types and editions were used. IQ data were not adjusted for floor effects [81, 82], since only a single individual had an IQ with a floored score (IQ = 40).

### Fibroblast culture

Fibroblast lines used for the current study were derived from biopsies performed over an 11-year period (2007–2018). The choice of dermal fibroblasts over peripheral blood cells was made because we have found the stability of the protein is greater during isolation from fibroblasts than for isolation from peripheral blood mononuclear cells (data not shown), presumably due to residual proteases in peripheral blood granulocytes and other lysed cell types or contributions from PBMCs that have been damaged during the freezing process. The constancy of the mean FMRP levels among normal controls across this period indicated that there was no effect of the duration of storage on the FMRP levels produced following revival and culture. To provide an equal starting point for measurements of FMRP levels, cryopreserved fibroblast lines were thawed and expanded in 60:40 media until 70–80% confluent, at which time each line was frozen at 500,000 cells/ml. A 20-year-old control male fibroblast line was used as a fiducial for all FRET plates, and FMRP levels were stable throughout the years it was tested. Five aliquots of the fibroblast line at passage five were pooled and distributed over 10 T-175 flasks, followed by growth until cells were 80–90% confluent, at which time cells were pooled and frozen down at 500,000 cells/ml.

### Genotyping

Genomic DNA isolation was performed on cultured fibroblast cells using the Gentra Puregene genomic DNA purification kit (Qiagen, Redwood City, CA; catalog number 158389). One-hundred to 250 ng of DNA was used to genotype each sample using AmplideX® PCR/CE *FMR1* kit (Qiagen, Redwood City, CA; cat. no.158389). PCR fragments were sent for fragment analysis on the ABI 3130xl Capillary Electrophoresis Genetic Analyzer in the College of Biological Sciences UCDNA Sequencing Facility at UC Davis. Peaks were analyzed and sized using Peak Scanner 2 (Applied Biosystems; Foster City, CA).

### Western blot analysis

To analyze the point mutation case, 1016–15, (nominal concentrations of 8 μg and 16 μg total protein) was determined by bicinchoninic acid (BCA) assay (Pierce; Rockford, IL; cat. no. 23225) for each sample and was run on a Criterion™ TGX Any kD™ Precast Gel (BioRad; Hercules, CA; cat. no. 567–1125) alongside 5 μl of Chameleon Duo Pre-stained Protein Ladder (LI-COR; Lincoln, NE; cat. no. 928–60000) (marker) for 30 min at 25 mA and then 1.5 h at 150 V. Proteins were transferred to a nitrocellulose membrane at 30 V overnight at 4°C. The membrane was blocked in 1:1 PBS:Odyssey® Blocking Buffer (LI-COR; 927–40000) for 1 h at room temperature. Mouse anti-FMRP (Millipore; Burlington, MA; cat. no. MAB2160; Lot no. 2548166) primary antibody in blocking buffer, supplemented with 0.1% Tween-20, was applied to the membrane at 1:5,000 overnight at 4°C. Subsequently, IRDye® 800CW Goat anti-Mouse (LI-COR; cat. no. 926–32210; Lot no. C50316-03) secondary antibody in blocking buffer, supplemented with 0.1% Tween-20, was applied to the membrane at 1:20,000 for 1.5 h at room temperature. Membrane was visualized on the LI-COR Odyssey Infrared Imager.

### FRET analysis

Fibroblasts (500,000 cells/ml) were thawed and seeded overnight at 100,000 cells/well into 24-well plates with RPMI 1640. The cells were grown overnight in 5% CO_2_, ambient O_2_. At the 24-h mark, samples were rinsed with Dulbecco's phosphate-buffered saline and lysed directly on the plate with 50 μl 1X Cisbio Human FMRP assay lysis buffer + Roche cOmplete^TM^ Ultra Protease Inhibitor Tablets (MilliporeSigma, Burlington, MA) for 2 h while rocking. FMRP quantification utilized the Cisbio Human FMRP assay (63ADK038PEC0; Anti-FMRP-K Lot no. 110514K; Anti-FMRP-d2; Lot no. 110514D; CisbioUS, Bedford, MA) and followed the manufacturer’s protocol. The assay uses homogeneous time-resolved fluorescence technology, as developed for quantification of FMRP [69, 79, 83]. The method uses two FMRP-specific monoclonal antibodies conjugated to fluorescent dyes; the donor was labeled with Eu^2+^-Cryptate and an acceptor designated d2. The time-resolved aspect of the assay exploits the long fluorescence decay times of the Eu^2+^-Cryptate donor fluor, such that FRET measurements do not occur until all sources of short-fluorescence-lifetime fluorescence (including from direct excitation of the acceptor) have decayed. Ten microliters of protein lysate were used in either triplicate or quadruplicate in a 384-well Opti-Plate (Perkin Elmer, Boston, MA), plus 10 μL homogeneous time-resolved fluorescence technology pre-mixed antibodies. Samples were incubated overnight in the dark and then read on the PerkinElmer VictorX5. Total protein concentrations were determined using the Thermo Fisher Micro BCA Assay (Thermo Fisher; Waltham, MA; cat. no. 23235). FMRP values were determined from interpolating FRET expression on a standard curve using a fiducial cell line. The BCA values were used to determine the ratio of interpolated FMRP to total protein. Finally, we have assessed the accuracy of the measurements by analyzing the coefficient of variation (CV) for each FMRP determination [CV (%) = 100 x σ/FMRP] (S1 Fig). For FMRP values above 20% of the mean FMRP among fibroblast lines with normal CGG repeat alleles, the CV is generally less than 20%; for FMRP levels from 1 to 10% of the normal mean level, the CV values range from ~20–100%. That is, for an FMRP level of 2%, a CV of 50% would represent a range of 1–3% for FMRP.

### Statistical analysis

All statistical analyses of data were conducted using SAS 9.4 (SAS Institute, Cary, NC). Results were expressed as mean ± standard deviation of mean. Group differences in means for quantitative measures were determined by analysis of variance (ANOVA) by sex. Proportions were compared between groups using Fisher’s exact test. Correlations of CGG-repeat size and FMRP level with IQ measures were assessed with Pearson’s correlation. Trend analyses of the relationship of IQ against FMRP level were conducted using a locally weighted regression (LOESS) and a piecewise linear regression, with adjustment for age and sex as needed. LOESS is a nonparametric regression technique that smooths the dependent variable (IQ) in a moving fashion and requires nonlinearity assumptions that are typical for conventional linear regression methods [84]. The LOESS smoothing parameter was inspected to identify the value, which minimized error but did not overfit the data. The LOESS smoothing parameter incorporates both the tightness of the fit and model complexity. Root mean square error (RMSE) and residuals were calculated for the LOESS fit across the entire data range. After LOESS fit was performed, the LOESS smoothing curve was inspected to identify possible change points for a piecewise linear regression through recursive partitioning to split data into two homogenous subsets based on a given change point. This process was to minimize the RMSE with the selected smoothing curve and was applied to each IQ measure. The final decision regarding which change point for a piecewise linear model should be applied to each IQ measure was made with consideration given to both the total RMSE and physical fit. The final piecewise linear regression chosen for each IQ measure was selected by considering the total RMSE, the best fit of the curve, and the visual appeal of the combined LOESS and piecewise linear regression curves. Two-tailed P-values less than 0.05 were considered statistically significant as appropriate.

## Results

### Demographics of the individuals providing dermal fibroblast samples for the current study

**Fig 1:**
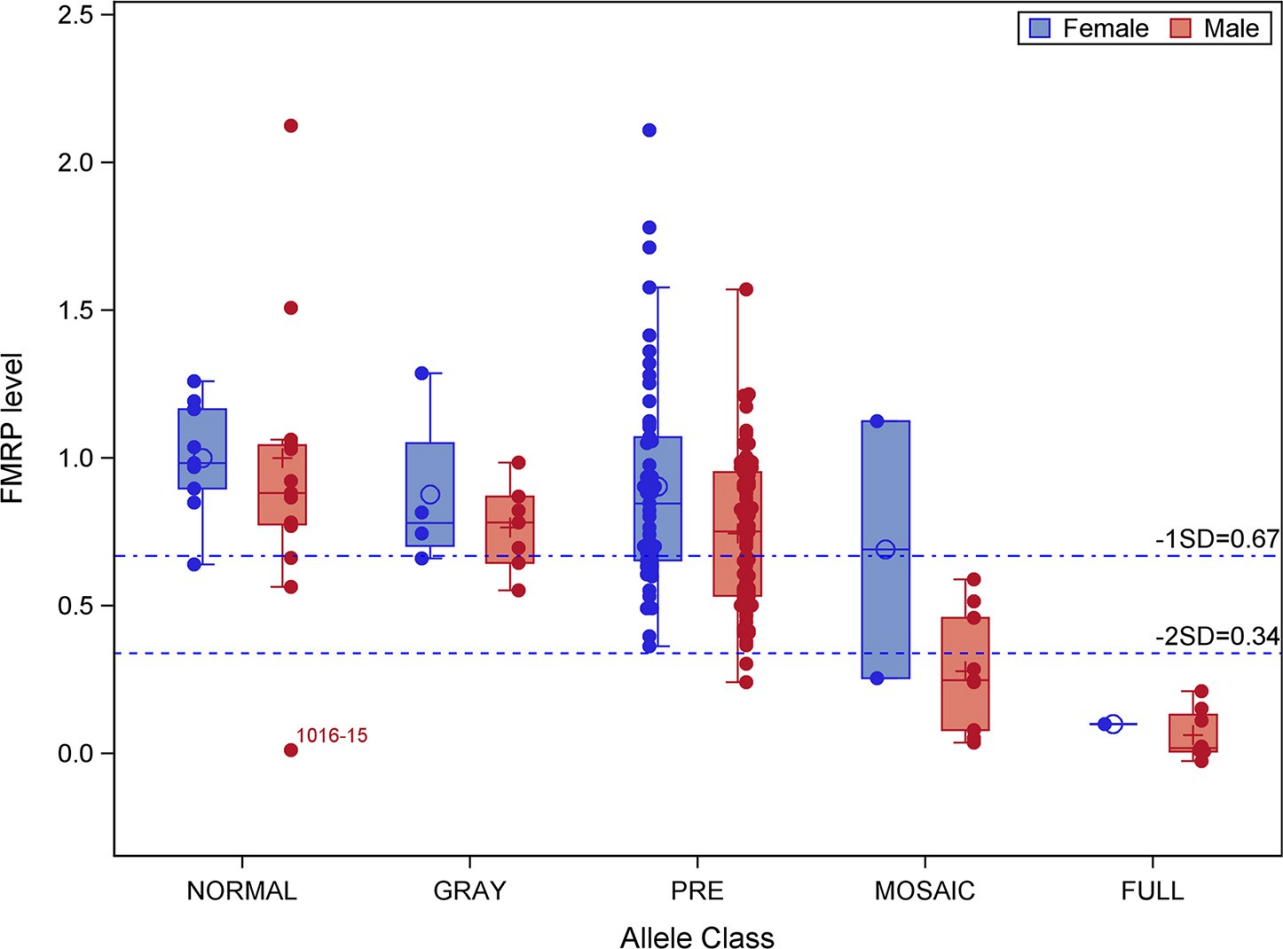
Distribution of FMRP levels within each allele class. Dashed lines indicate the values of FMRP 1 SD and 2 SD (as indicated) below the relative mean level (= 1.0) of those with normal CGG-repeat alleles (see, also: Fig 2). Individual 1016–15 has a normal CGG repeat (21 CGG) but has a point mutation within the FMRP coding region [80] that prevented detection by the FRET assay (See: S2 Fig).

A total of 195 fibroblast samples were cultured from punch skin biopsies from adults (74 females and 121 males). Eleven cases, with secondary known genetic neurodevelopmental disorders, a point mutation in one case, and/or dysmorphic features atypical for FXS but with normal FMRP levels, were subsequently excluded from statistical analyses, as previously mentioned. Descriptive statistics of the 184 participants used for this present study are presented in Table 1. One of the 11 excluded cases (1016–15), a severely affected ten-year-old male [Full scale IQ (FSIQ), 40; Performance (PIQ), 42; Verbal IQ (VIQ), 43] with a point mutation and a normal CGG repeat [80], is displayed in Fig 1 but was excluded from regression analyses because the point mutation prevented detection of the protein using the FRET assay.

**Table 1:** Descriptive statistics of patient characteristics, by sex.

Variable	All	Females	Males	P-value*
N (%)	184	72 (39%)	112 (61%)	
Age	46.98 ± 21.88	46.92 ± 19.92	47.02 ± 23.15	0.9757
CGG-repeat size	90.68 ± 65.13	76.41 ± 37.18	100.14 ± 77.09	0.0185
Allele class, N				0.2429
Normal	22	9	13	
Gray	11	4	7	
Pre	130	55	75	
Mosaic	11	2	9	
Full	9	1	8	
Missing	1	1	0	
PIQ	100.22 ± 19.60	103.23 ± 16.95	98.28 ± 20.98	0.0976
VIQ	108.07 ± 21.15	109.21 ± 17.30	107.33 ± 23.56	0.5594
FSIQ	102.71 ± 21.86	105.11 ± 17.87	101.16 ± 24.03	0.2326

* Significance of difference between males vs. females.

**Fig 2:**
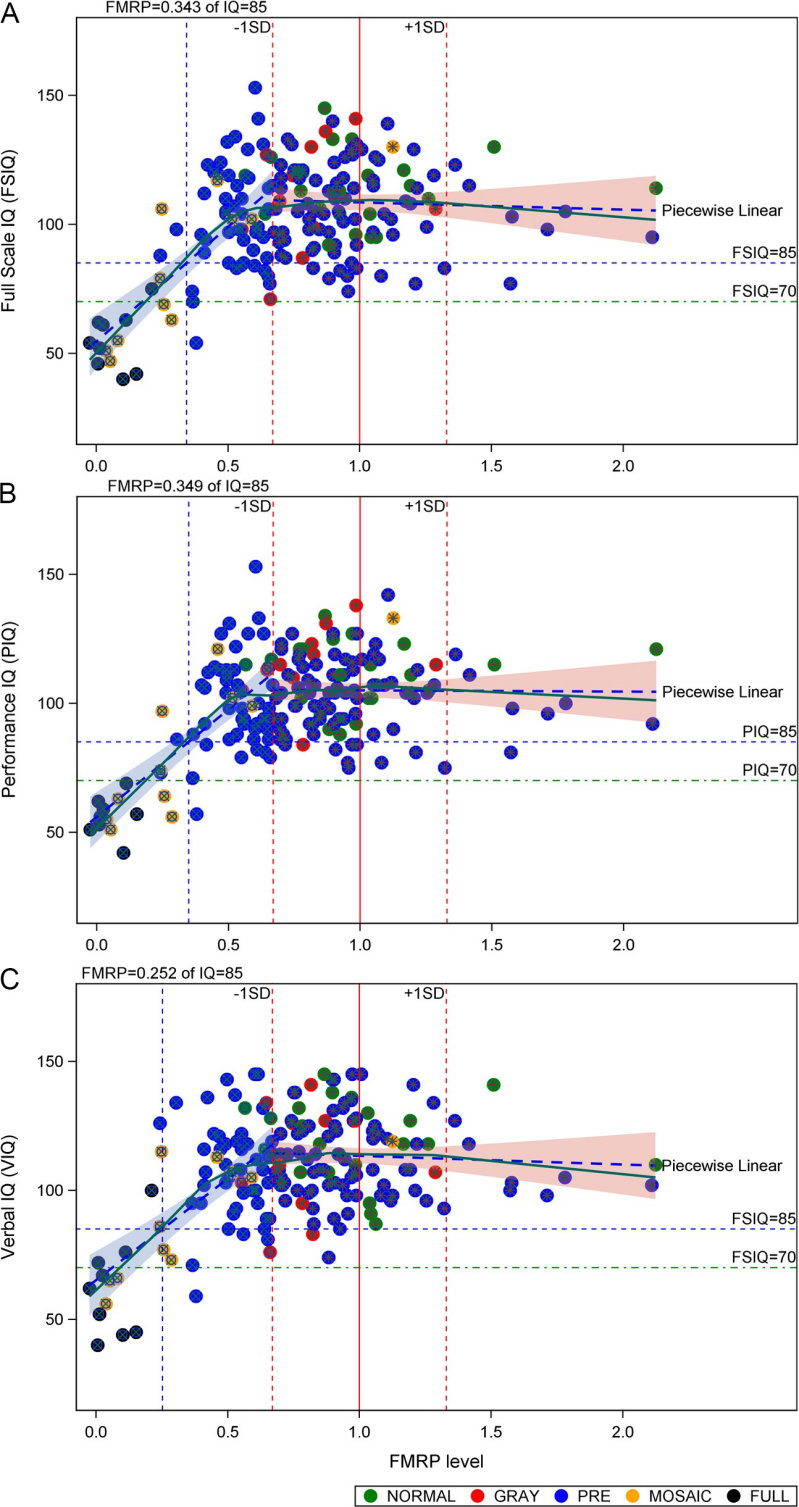
Dependence of IQ on FMRP levels in cultured dermal fibroblasts, accounting for sex as a covariate. (**A**) FSIQ, (**B**) PIQ, and (**C**) VIQ were determined using age-appropriate instruments as delineated in the Methods section. FMRP levels were determined using FRET. All FMRP levels were normalized to the mean value of FMRP levels (= 1.0) among individuals with normal CGG repeats, excluding the *FMR1* point mutation (1016–15). Data displayed are for both males and females since there is no significant differential dependence of the regression lines on sex (P > 0.05, see S1 Table); separate regression analyses for males and for females are displayed in S3A–S3F Fig. Symbols specify allele classes as indicated. Plus/minus 1 SD for FMRP levels are indicated as red vertical dashed lines. Lower limit of normal IQ (= 85) and borderline IQ (= 70) are indicated as horizontal blue and green dashed lines, respectively. The FMRP level at which the regression line for IQ passes 85 is indicated by a vertical (blue) dashed line.

The distributions of FMRP levels across allele classes are displayed in Fig 1, where all FMRP values for normal or gray-zone (~46–54 CGG repeats) alleles for the current, relatively small dataset, are well above the threshold value for the normal full-scale IQ (FSIQ) range; the sole exception is a normal CGG-repeat allele that harbors a point mutation in the FMRP coding sequence [80]. This case is interesting in that the FRET assay is unable to detect the mutant protein even though the protein is produced at normal levels by Western (S2 Fig). A much broader distribution is evident among PM alleles, where larger alleles are associated with lower FMRP levels (correlation coefficient R = -0.22, P = 0.0103). FM alleles are all associated with FMRP levels below the threshold level corresponding to FSIQ = 85 in the fitting model represented in Fig 2, in keeping with the general observation that all males with FM alleles have some degree of cognitive impairment. An important, albeit less well-defined, allele class is that comprising mosaic alleles, since individuals within this class often have multiple alleles with variable proportions in PM and FM ranges, and with variable degrees of methylation of individual alleles (Fig 1).

### The FMRP-dependence of IQ is piecewise linear, with a threshold value for FMRP-dependence approximately one SD below the normal mean level

For the current dataset, the transition from a strong dependence of IQ on FMRP to its independence of FMRP levels occurs at a value of FMRP approximately 1 SD below its mean value among individuals harboring normal CGG-repeat alleles (Fig 2). This relationship holds for both Verbal and Performance IQs (VIQ, PIQ), as well as for FSIQ. Moreover, this piecewise linear behavior remained intact even after adjusted for age and sex and in sex-specific analyses (S3 Fig and S2 Table). For participants with FMRP levels below the -1 SD inflection point, IQ measures steeply decrease with further decreases of FMRP levels. An important feature of this dataset is that the regression line for IQ passes the lower end of the normal IQ range (IQ = 85) at approximately 2 SD below the normal mean FMRP level and passes the cut-off for intellectual disability (IQ = 70) at approximately 2.5 SD below the normal mean level of FMRP (Fig 2 and Table 2).

**Table 2:** Piecewise regression models assessing the relationships between X = FMRP level (normalized to normal controls) and Y = IQ, and estimated FMRP levels corresponding to IQ = 85 and 70.

IQ Measure	FMRP below -1SD					FMRP above -1SD		
	Fitted Regression Model	P-value	R^2^ value	Estimated FMRP levels		Fitted Regression Model	P-value	R^2^ value
				IQ = 70	IQ = 85			
Full Scale IQ	*FSIQ = 54*.*48 + 88*.*97 FMRP*	< 0.0001	0.481	0.174	0.343	*FSIQ = 111*.*24–2*.*78 FMRP*	0.6326	0.0021
Performance IQ	*PIQ = 55*.*90 + 83*.*32 FMRP*	< 0.0001	0.5063	0.169	0.349	*PIQ = 105*.*45–0*.*48 FMRP*	0.9274	0.0001
Verbal IQ	*VIQ = 65*.*13 + 78*.*68 FMRP*	<0.0001	0.3965	0.062	0.252	*VIQ = 116*.*72–3*.*36 FMRP*	0.5502	0.0033

## Discussion

In the current study, we quantified levels of FMRP in a cohort of 184 subjects using the method of homogeneous, time-resolved FRET [83] and have related these levels to age-appropriate IQ measures. We found that the distribution of relative FMRP levels among normal controls is broadly similar to equivalent distributions determined in platelets [77] and in dried blood spots [78]. Two features of the FMRP–IQ relationship are noteworthy. First, there is a threshold level of FMRP at approximately 70% (-1 SD) of the mean FMRP level for those with normal CGG-repeat alleles, above which there is no further dependence of IQ on FMRP levels—that is, a potential state of sufficiency such that further increases above the threshold do not relate to further cognitive gain. Thus, based on this cross-sectional analysis, attempts to further increase FMRP in normal individuals would not be expected to yield changes in cognitive processes underlying IQ. Indeed, further increases in FMRP well beyond the normal range may lead to behavioral dysfunction, as demonstrated in animal models [85, 86]. Second, for FMRP levels below the threshold, there is a strong, positive relationship between FMRP levels and cognitive abilities. Although the number of cases in this range is small in the current study, the approximate linear relationship suggests that an FMRP level of only about one-third of the normal mean level is sufficient to support a mean IQ level at the lower end of the normal IQ range (= 85), and that an FMRP level of only 10–20% of normal would support a mean IQ at the borderline level. In aggregate, these observations support the idea that even relatively modest increases in FMRP, as obtained through efforts to induce its expression, could have a beneficial effect on cognitive outcomes. Similar conclusions have been drawn following introduction of *FMR1* transgenes into *Fmr1* knockout mice for both audiogenic seizure activity [87] and for motor/behavioral functions [88].

A further implication of the current work is the expectation of the range of IQs within the FM range. Among the eight males that harbored FM alleles without evidence of smaller PM alleles, FMRP levels ranged from 0 to 21% relative of the normal mean FMRP level. The 21% level was associated with a relatively small FM allele (250 CGG repeats) and a borderline IQ (FSIQ = 70). The remaining individuals (FMRP = 0–15%) all had FSIQs ranging from 40 to 62. Given the small number of individuals with FM alleles, these results must be viewed as provisional; however, they should serve to underscore the need for careful determination of both FMRP levels and genotype before declaring FM alleles to be associated with IQ levels in the normal range. IQs in the normal range for apparent FM alleles are actually more likely reflective of cryptic mosaicism, where–depending on the nature of size and methylation mosaicism–IQs can reach or exceed the lower level of the normal IQ range (Fig 1). This complex issue will require larger studies of both mosaic and FM cases to establish a more quantitative relationship between allele type and IQ, since the level of FMRP will depend not only on the size range for multiple alleles but also the relative abundance of each allele, as well as their separate extents of methylation. Absent such studies, we feel it more appropriate to establish the relationship between FMRP, which reflects the accumulated effects of allele types, and IQ.

The observed relationship between FMRP and IQ below the -1 SD threshold may have implications for the general population. Our observations tentatively suggest that FMRP levels would need to be reduced to about -2 SD from the normal mean (~34%) to have an average IQ at the lower boundary of the normal IQ range (IQ = 85). Thus, were our current observations to hold for much larger cohorts, we would predict that about 2–3% of the general population would have an IQ below 85 as a consequence of lowered FMRP. Of course, there are many causes of intellectual disability other than FXS, but we do not know the relationship of FMRP to intellectual deficit in most of the other disorders. Admittedly, these extrapolated predictions are highly provisional since they are based on a very small dataset; they should be viewed primarily from the standpoint of encouraging larger studies. However, two points follow from our observations. First, Bernard et al. [89] demonstrated in a normal rat model that early life seizures reduce dendritic FMRP at/near synapses, with a corresponding shift of FMRP to the neuronal soma, despite an apparent increase in overall hippocampal FMRP [90]. Thus, the occurrence of early-life seizure creates a functional deficit of FMRP for synaptic functioning in the rat, with the corresponding reduction of cognitive function. For a normal child, a single seizure is not likely to substantially reduce cognitive strength, though in wildtype mice, single seizures have been demonstrated to affect long-term cognitive outcomes [91]; however, for a child whose FMRP levels are already low, early-life seizures could have a profound effect on cognitive function, underscoring the need for aggressive treatment of seizure activity in a child with fragile X syndrome or PM involvement. The second, broader implication of the current study concerns the reports of reduced levels of FMRP in individuals with major psychiatric disorders, where FMRP levels down to ~25% of normal have been observed in individuals with schizophrenia [73, 74]. The critical question for this latter instance is whether intrinsically low FMRP levels predispose to major psychiatric disorders or whether the processes involved with those disorders drive a reduction in FMRP. Resolution of this issue will have important ramifications for treatment of both psychiatric disorders and for fragile X syndrome.

In one of the most comprehensive analysis of the association between FMRP and IQ (derived from the Wechsler scales) in 290 individuals with full or PM alleles, Loesch et al. [92] utilized the immunocytochemical staining method of Willemsen et al. [59], which uses the fraction of lymphocytes staining positive for FMRP as a surrogate measure for relative FMRP concentration. As noted by Willemsen et al. [59], whereas this approach is useful for distinguishing individuals with FXS who do not produce FMRP from those with FMRP levels in the normal range (cf. Fig 1B of [92]), the staining method does not measure relative FMRP levels *per se*. Not surprisingly, therefore, Loesch et al. [92] observed a very large variance in the percent of FMRP-positive cells at all IQ levels, ranging from approximately 0–55% at IQ = 40 to 50–95% at IQ = 100, which diminished the portion of the variance that could be attributed to a dependence of cognitive measures on FMRP. Many FM alleles reported in this and previous studies are likely to be cryptic mosaics [58], where broad distributions of alleles may remain undetected, but which in aggregate would contribute to FMRP production.

Several limitations are inherent in the current study, foremost among them being the relatively small size of the sample cohort. While we believe that the general conclusions regarding the relationship between FMRP and IQ are sound, our observations and interpretations underscore the need for much larger sample cohorts. In this regard, because individuals were recruited through ongoing studies of families with fragile X syndrome and associated disorders, there is always the possibility of unrecognized bias both in the subjects with fragile X PM and FM alleles, and also in terms of the characteristics of controls, some of whom are unaffected (normal *FMR1* gene) family members. We note in this regard that mean IQ values for the normal controls are generally well above 100. Due to the retrospective nature of the study and wide age range of participants, data from several different IQ tests were used. Although these tests utilize the same scoring metric for standardization (mean = 100, SD = 15) and measure general intelligence, they vary in their coverage of subdomains of intelligence and rely on different normative samples, and therefore systematic bias in scores is possible across tests. This bias may have introduced some unwanted error in our analyses. Studies utilizing the same test across all participants would likely yield stronger correlations between FMRP and IQ scores.

Also, given that the heritability of intelligence in the general population is as high as 80% [93], an important limitation of this and other studies examining the association between FMRP and IQ is that they have generally not accounted for factors such as parental IQ or shared environmental factors. For example, while a person with modest but significant reduction in FMRP may have an IQ in the broad normal range, this level of cognitive function may be very substantially below what is expected based on mean parental IQ [42]. Dyer-Friedman et al. [94] demonstrated that mean parental IQ was significantly associated with offspring IQ in both normal siblings and females with the FM but not in males with the FM, possibly indicating that the background environmental and genetic effects on IQ are stronger among those with less severe deficits and more variable FMRP levels. Thus, future studies seeking to obtain better estimates of the effects of the FMRP deficit should account for these shared factors if possible.

Another fundamental limitation of the current work, as with many other studies of CNS-based phenotypes (e.g., the relationship between IQ and brain FMRP levels), is the necessity of using a peripheral measure of relative FMRP level (normalized to mean FMRP among those with normal CGG repeats) as a surrogate for the brain relative FMRP level. This approach is based on the lack of accessibility of the CNS measures and thus assumes that the relative peripheral and brain FMRP levels have the same dependence on CGG repeat, and that the CGG repeat and methylation status are comparable in peripheral and brain tissues; this latter assumption is the basis for clinical testing for fragile X-associated disorders and is likely to be generally but not universally true. One approach to evaluate the validity of this assumption is to assess the magnitude of the residuals for IQ as a function of relative FMRP level. For FMRP levels that are greater than -1 SD (Fig 2), the IQ distribution becomes independent of FMRP level, and the residuals thus depend on other factors (e.g., background gene effects, environmental factors); however, for further decreases in FMRP below -1 SD, where IQ is strongly dependent on FMRP level, substantial differences in peripheral and brain FMRP levels would be expected to result in larger IQ residuals. We have analyzed this issue by plotting the residuals (S4 Fig) as a function of FMRP, demonstrating that there is no evidence of an increase in the residuals for IQ. This observation suggests that, at least as a first approximation, the use of peripheral FMRP measures to gauge IQ is reasonable.

Finally, the potential implications we describe regarding whether therapeutically driven changes in FMRP may or may not contribute to cognitive changes at different levels of FMRP expression are based upon cross-sectional data; longitudinal data collected from individuals over time would be needed to test the validity of these interpretations.

## Supporting information

S1 Fig Plot of the coefficient of variation (CV, %) as a function of the measurement-mean FMRP level determined for each fibroblast sample by FRET. FMRP levels are normalized to the groupwise mean FMRP level for fibroblasts with normal CGG repeats (designated FMRP *norm*,*ctrl*).
(TIF)

S2 Fig FMRP is present in normal concentrations in a fibroblast patient sample with an *FMR1* point mutation. Western blot analysis of the expression of FMRP in fibroblast lines from male patients with a control allele (**Control**), control allele with a point mutation (**Point**), or FM allele (**Full**). The control sample is the fiducial used for FRET plates in this study. The point mutation sample is 1016–15.
(TIF)

S3 Fig Dependence of IQ on FMRP levels in cultured dermal fibroblasts, separated by sex. Females: (**A**) FSIQ, (**B**) PIQ, and (**C**) VIQ; Males: (**D**) FSIQ, (**E**) PIQ, and (**F**) VIQ. IQs were determined using age-appropriate instruments as delineated in the Methods section. FMRP levels were determined using FRET. All FMRP levels were normalized to the mean value of FMRP levels (= 1.0) among individuals with normal CGG repeats (<45 CGG repeats), excluding the *FMR1* point mutation (1016–15). Symbols specify allele classes as indicated. Plus/minus 1 SD for FMRP levels are indicated as red vertical dashed lines. Lower limit of normal IQ (= 85) and borderline IQ (= 70) indicated as horizontal blue and green dashed lines, respectively. The FMRP level at which the regression line for IQ passes 85 is indicated by a vertical (blue) dashed line.
(TIF)

S4 Fig Absence of any increase in dispersion of IQ residuals for FMRP levels below -1 SD from the mean FMRP level among normal CGG-repeat controls. (A) Males only; (B) both males and females.
(TIF)

S1 Table Gender-specific piecewise regression models assessing the relationships between X = FMRP level (normalized to normal controls) and Y = IQ. (DOCX)

S2 Table Restrict to normal controls: Piecewise regression models assessing the relationships between X = FMRP level and Y = subject IQ. (DOCX)

S1 Raw Images Original Western blot for S2 Fig. Western blot analysis of the expression of FMRP in fibroblast lines from male patients with a control allele (**Control**), control allele with a point mutation (**Point**), or FM allele (**Full**). The control sample is the fiducial used for FRET plates in this study. The point mutation sample is 1016–15. Unused wells are marked with an **“X**”. Raw image was captured using Near-infrared (NIR) fluorescence on LI-COR Odyssey (800 nm channel).
(TIF)
