# Supplementary figures and images for: Association between IQ and FMR1 protein (FMRP) across the spectrum of CGG repeat expansions
Source: PLoS One. 2019 Dec 31;14(12):e0226811. doi: 10.1371/journal.pone.0226811 (PMC6938341; doi:10.1371/journal.pone.0226811)

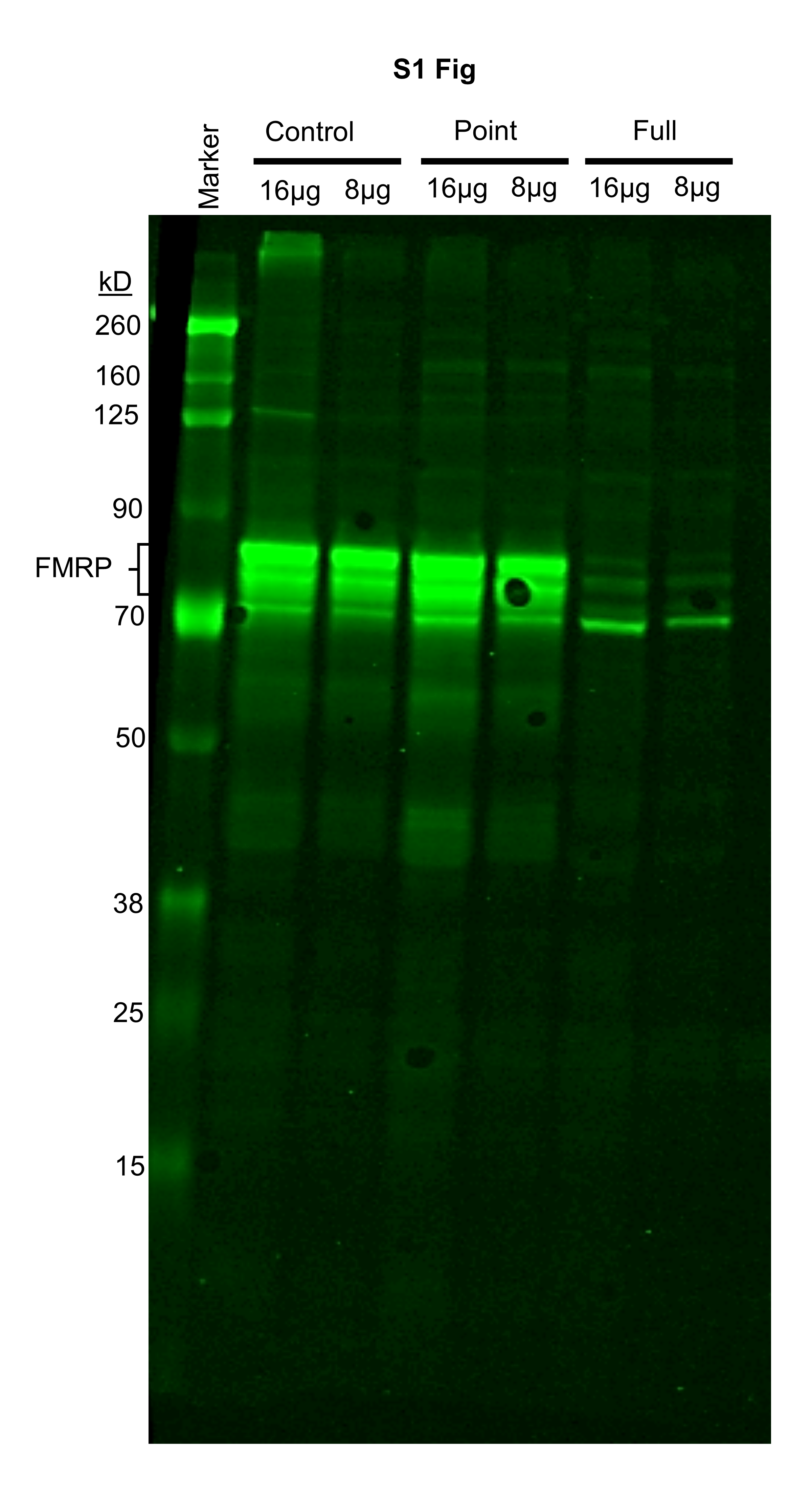

Supplement: S2 Fig — Western blot analysis of the expression of FMRP in fibroblast lines from male patients with a control allele (Control), control allele with a point mutation (Point), or FM allele (Full). The control sample is the fiducial used for FRET plates in this study. The point mutation sample is 1016–15. (TIF) [file pone.0226811.s002.tif]

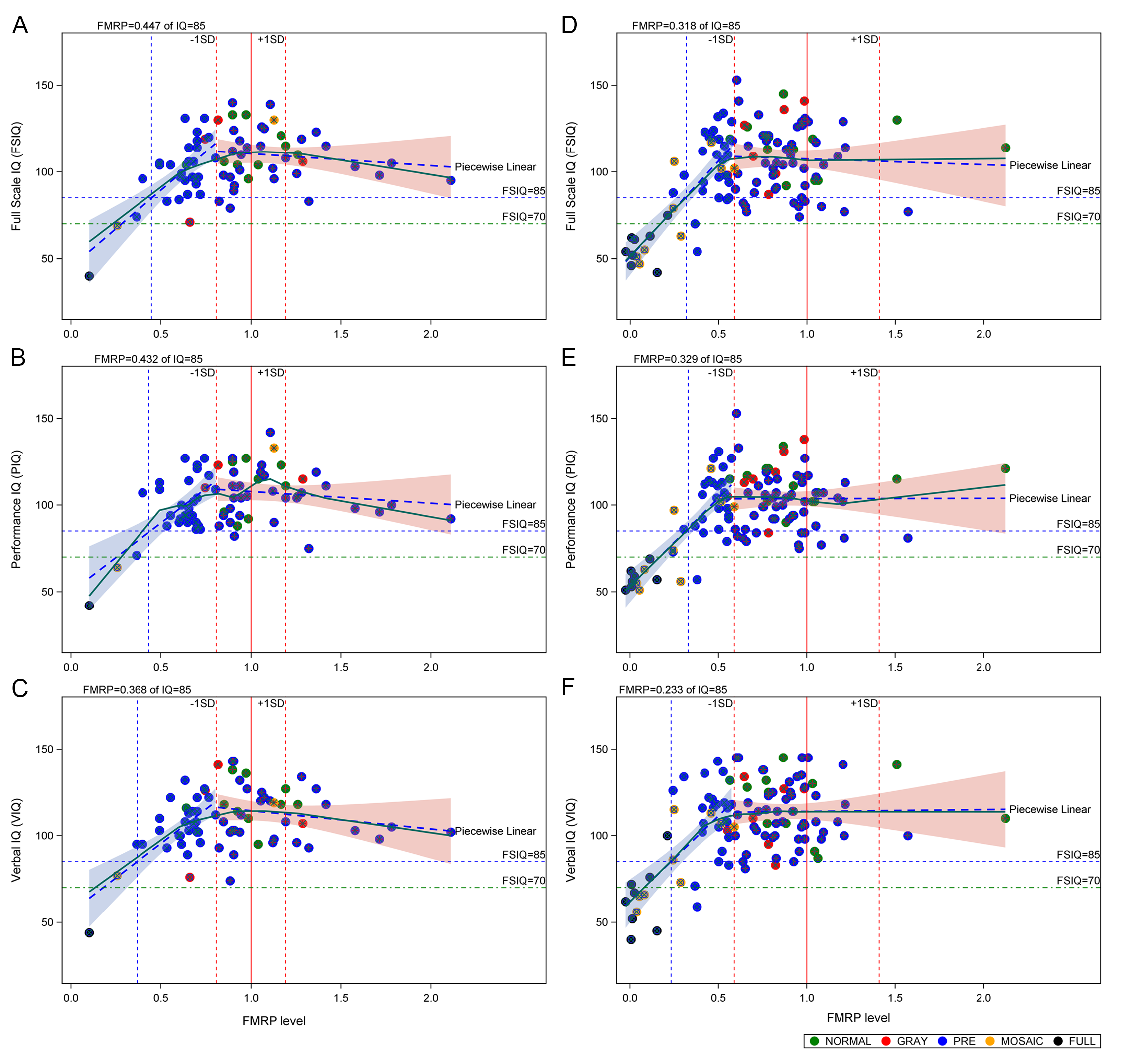

Supplement: S3 Fig — Females: (A) FSIQ, (B) PIQ, and (C) VIQ; Males: (D) FSIQ, (E) PIQ, and (F) VIQ. IQs were determined using age-appropriate instruments as delineated in the Methods section. FMRP levels were determined using FRET. All FMRP levels were normalized to the mean value of FMRP levels (= 1.0) among individuals with normal CGG repeats (<45 CGG repeats), excluding the FMR1 point mutation (1016–15). Symbols specify allele classes as indicated. Plus/minus 1 SD for FMRP levels are indicated as red vertical dashed lines. Lower limit of normal IQ (= 85) and borderline IQ (= 70) indicated as horizontal blue and green dashed lines, respectively. The FMRP level at which the regression line for IQ passes 85 is indicated by a vertical (blue) dashed line. (TIF) [file pone.0226811.s003.tif]

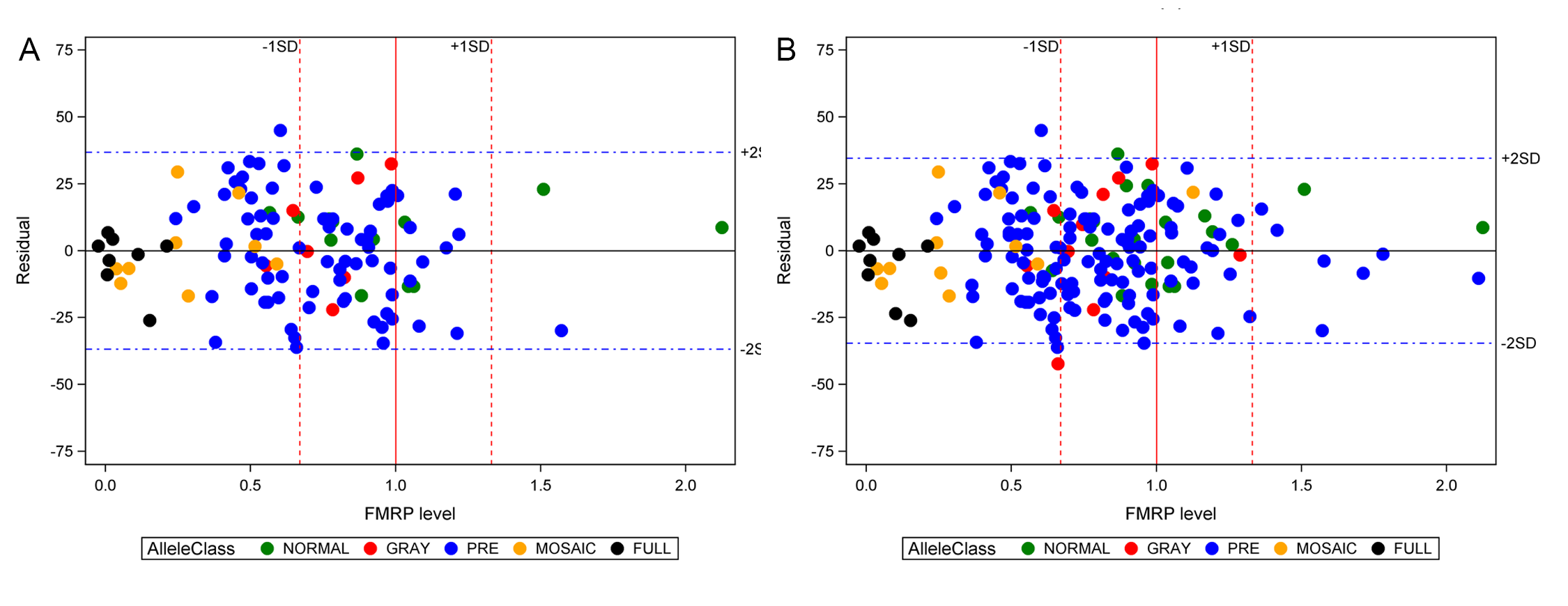

Supplement: S4 Fig — (A) Males only; (B) both males and females. (TIF) [file pone.0226811.s004.tif]

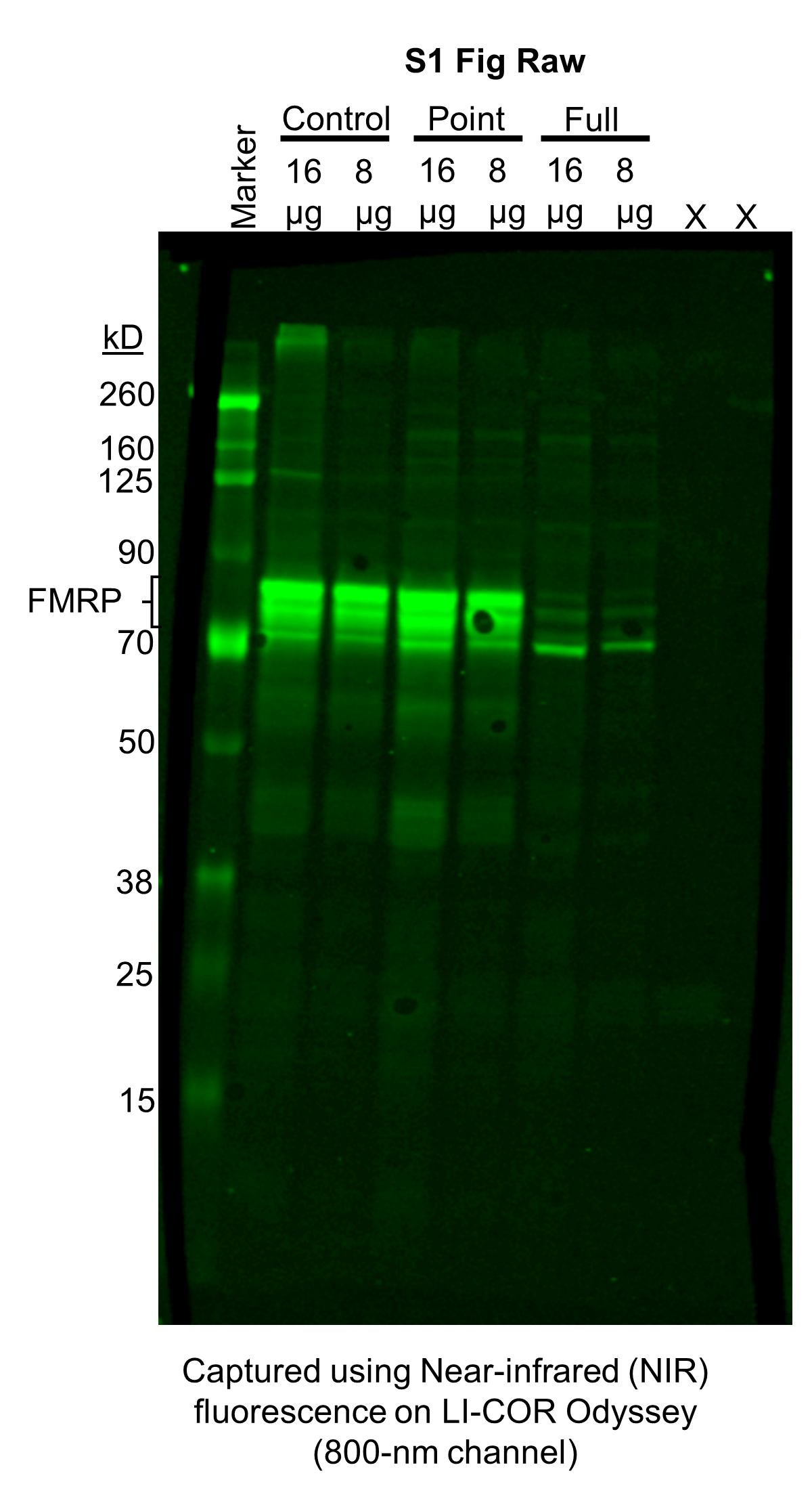

Supplement: S1 Raw Images — Western blot analysis of the expression of FMRP in fibroblast lines from male patients with a control allele (Control), control allele with a point mutation (Point), or FM allele (Full). The control sample is the fiducial used for FRET plates in this study. The point mutation sample is 1016–15. Unused wells are marked with an “X”. Raw image was captured using Near-infrared (NIR) fluorescence on LI-COR Odyssey (800 nm channel). (TIF) [file pone.0226811.s007.tif]
