# Supplementary material for: Association between IQ and FMR1 protein (FMRP) across the spectrum of CGG repeat expansions
Source: PLoS One. 2019 Dec 31;14(12):e0226811. doi: 10.1371/journal.pone.0226811 (PMC6938341; doi:10.1371/journal.pone.0226811)
Supplement: S1 Table — (DOCX) [file pone.0226811.s005.docx]

**S1 Table. Gender-specific piecewise regression models assessing the relationships between X = FMRP level (normalized to normal controls) and Y = IQ.**

| **IQ Measure** | **FMRP below -1SD** | | | | | **FMRP above -1SD** | | |
| --- | --- | --- | --- | --- | --- | --- | --- | --- |
|  | **Fitted Regression Model** | **P-value** | **R^2^ value** | **Estimated FMPR levels** | | **Fitted Regression Model** | **P-value** | **R^2^ value** |
|  |  |  |  | **IQ = 70** | **IQ = 85** |  |  |  |
| Full Scale IQ |  |  |  |  |  |  |  |  |
| Females | *FSIQ = 45.03 + 89.42 FMRP* | 0.0015 | 0.4756 | 0.279 | 0.447 | *FSIQ = 112.53 - 3.11 FMRP* | 0.6512 | 0.004 |
| Males | *FSIQ = 54.17 + 96.99 FMRP* | < 0.0001 | 0.5339 | 0.163 | 0.318 | *FSIQ = 110.88 - 3.34 FMRP* | 0.7438 | 0.002 |
| Sex Difference* |  | 0.931 |  |  |  |  | 0.1999 |  |
| Performance IQ |  |  |  |  |  |  |  |  |
| Females | *PIQ = 45.05 + 92.55 FMRP* | 0.0009 | 0.5304 | 0.27 | 0.432 | *PIQ = 108.24 - 1.87 FMRP* | 0.7831 | 0.0015 |
| Males | *PIQ = 56.52 + 86.47 FMRP* | < 0.0001 | 0.5283 | 0.156 | 0.329 | *PIQ = 103.57 + 0.14 FMRP* | 0.9867 | 0.000005 |
| Sex Difference* |  | 0.7508 |  |  |  |  | 0.2526 |  |
| Verbal IQ |  |  |  |  |  |  |  |  |
| Females | *VIQ = 54.49 + 82.89 FMRP* | 0.0027 | 0.4406 | 0.187 | 0.368 | *VIQ = 118.67 - 5.73 FMRP* | 0.4065 | 0.0133 |
| Males | *VIQ = 65.09 + 85.60 FMRP* | < 0.0001 | 0.4347 | 0.057 | 0.233 | *VIQ = 113.16 + 0.84 FMRP* | 0.9301 | 0.0001 |
| Sex Difference* |  | 0.9747 |  |  |  |  | 0.5555 |  |

*Sex difference indicates whether the relationship between FMRP level and a given IQ measure was statistically different between males and females.
