# Supplementary material for: Association between IQ and FMR1 protein (FMRP) across the spectrum of CGG repeat expansions
Source: PLoS One. 2019 Dec 31;14(12):e0226811. doi: 10.1371/journal.pone.0226811 (PMC6938341; doi:10.1371/journal.pone.0226811)
Supplement: S2 Table — (DOCX) [file pone.0226811.s006.docx]

**S2 Table. Restrict to normal controls: Piecewise regression models assessing the relationships between X = FMRP level and Y = subject IQ.**

|  | **FMRP below -1SD** | | | **FMRP above -1SD** | | |
| --- | --- | --- | --- | --- | --- | --- |
| **IQ Measure** | **Fitted Regression Model** | **P-value** | **R^2^ value** | **Fitted Regression Model** | **P-value** | **R^2^ value** |
| *Overall* | No observation with FMRP levels below -1SD | | |  |  |  |
| Full Scale IQ |  |  |  | *FSIQ = 114.18 + 0.28 FMRP* | 0.9802 | 0.00004 |
| Performance IQ |  |  |  | *PIQ = 105.83 + 5.43 FMRP* | 0.5701 | 0.0194 |
| Verbal IQ |  |  |  | *VIQ = 122.04 - 3.47 FMRP* | 0.789 | 0.0043 |
| *Female* |  |  |  |  |  |  |
| Full Scale IQ |  |  |  | *FSIQ = 124.11 - 8.96 FMRP* | 0.817 | 0.0096 |
| Performance IQ |  |  |  | *PIQ = 105.20 + 6.27 FMRP* | 0.8694 | 0.0049 |
| Verbal IQ |  |  |  | *VIQ = 135.92 - 15.23 FMRP* | 0.7032 | 0.0259 |
| *Male* |  |  |  |  |  |  |
| Full Scale IQ |  |  |  | *FSIQ = 113.03 + 1.16 FMRP* | 0.9308 | 0.0009 |
| Performance IQ |  |  |  | *PIQ = 105.69 + 5.38 FMRP* | 0.603 | 0.0313 |
| Verbal IQ |  |  |  | *VIQ = 119.53 - 2.20 FMRP* | 0.8911 | 0.0022 |
